# Phytochemical Analysis and Anti-Inflammatory Activity of the Extracts of the African Medicinal Plant *Ximenia caffra*

**DOI:** 10.1155/2015/948262

**Published:** 2015-02-17

**Authors:** Jing Zhen, Yue Guo, Tom Villani, Steve Carr, Thomas Brendler, Davis R. Mumbengegwi, Ah-Ng Tony Kong, James E. Simon, Qingli Wu

**Affiliations:** ^1^New Use Agriculture and Natural Plant Products Program, Department of Plant Biology and Pathology, Rutgers University, 59 Dudley Road, New Brunswick, NJ 08901, USA; ^2^Department of Medicinal Chemistry, Ernest Mario School of Pharmacy, Rutgers University, 160 Frelinghuysen Road, Piscataway, NJ 08854, USA; ^3^Department of Pharmaceutics, Ernest Mario School of Pharmacy, Rutgers University, 160 Frelinghuysen Road, Piscataway, NJ 08854, USA; ^4^National Botanical Research Institute, Windhoek, Namibia; ^5^PlantaPhile, 705B Park Avenue, Collingswood, NJ 08108, USA; ^6^Drug Discovery and Development Program, Science, Technology and Innovation Division, Multidisciplinary Research Center University of Namibia, 340 Mandume Ndemufayo Avenue Private Bag Box 13301, Windhoek, Namibia

## Abstract

A method was developed for identification and quantification of polyphenols in the leaves of *Ximenia caffra* using HPLC/UV/MS. Based on analyzing the MS and UV data and in comparison to the authentic standards, a total of 10 polyphenols were identified and quantified, including gallic acid, catechin, quercetin, kaempferol, and their derivatives. The total content of these compounds was found to be approximately 19.45 mg/g in the leaf and the most abundant is quercetin-rutinoside (9.08 mg/g). The total phenolic content as measured by Folin-Ciocalteu assay was 261.87 ± 7.11 mg GAE/g and the total antioxidant capacity as measured *in vitro* was 1.46 ± 0.01 mmol Trolox/g. The antiproliferative effect of the leaf extract was measured by MTS assay with IC_50_ value of 239.0 ± 44.5 *μ*g/mL. Cell-based assays show that the leaf extract inhibits the mRNA expression of proinflammatory genes (IL-6, iNOS, and TNF-*α*) by using RT-qPCR, implying its anti-inflammatory effects. It was further demonstrated that the underlying therapeutic mechanism involves the suppression of NF-*κ*B, a shared pathway between cell death and inflammation.

## 1. Introduction

*Ximenia caffra,* also known as “large sourplum,” is a member of a genus of flowering plants in the Olacaceae family, which grows in the southern African region [1, 2].* X. caffra* has been used in foods and traditional medicine. Its fruit, considered to be rich in vitamin C, potassium, and protein, has been commonly added into porridges and made into jam [3, 4]. The dried seed of* X. caffra* contains a substantial quantity of unsaturated fatty acids; the most abundant is oleic acid [5]. As such, the extracted seed oil would be used as a feed ingredient and domestic biofuel [6]. As a traditional medicine, local herbalists have been using the leaves and root of* X. caffra* for treatment of wounds, infections, fever, infertility, and diarrhea [7–9]. Recent research in laboratory confirmed that the leaf and root extracts of* X. caffra* have antigonococcal, antibacterial, and antifungal activities, which corroborate with its traditional use very well [7, 8, 10, 11]. Compared with a number of researches on its pharmacological activities, only one paper has been published on the phytochemical composition analysis of* X. caffra* leaf, in which the total flavonoid content was only roughly estimated using vanillin assay [8]. Bioactive phytochemicals contained in the leaf of related species* X. americana*, from western and eastern sub-Sahara Africa, were investigated and led to the identification of sambunigrin, gallic acid, and quercetin, and the glycosides by spectrometric methods [12].

Most of the studies with* X. caffra* focus on the fruit and seed, as these represent the products of current commerce and traditional use, rather than the leaf. In this study, we purposefully investigate the phytochemical profile of* X. caffra* leaf, which has not been previously examined using LC/UV/MS. Interestingly though not unexpectedly, a number of polyphenol compounds were discovered in the leaf. Polyphenols are a class of compounds characterized by the presence of large multiples of phenol structural units and abundantly exist in food products, including crops, fruit, vegetables, tea, and wine [13–15]. These compounds have shown a wide range of biological activities including anti-inflammation and the prevention of cancer and cardiovascular diseases [16, 17].

Based upon the chemical profile determined and to further explore new applications for the leaves while extending the traditional uses, we investigated the leaves for their antioxidant activity, total phenolic content, and antiproliferation activity using the Folin-Ciocalteu assay, Trolox equivalent antioxidant capacity radical scavenging assay, and the MTS cell proliferation assay, respectively. Additionally, the effects of extracts on the mRNA expression of proinflammatory genes were assessed by RT-qPCR through the quantification of inducible nitric oxide synthetase (iNOS), interleukin 6 (IL-6), and tumor necrosis factor *α* (TNF-*α*) induced by lipopolysaccharide (LPS) in murine RAW 264.7 macrophage cells. Interleukin 6 (IL-6), a cytokine secreted by T cells and macrophages, is produced at the inflammation sites during infection and tissue damage. IL-6 has stimulatory effects on immune cells and can induce chronic inflammatory responses. Thus, targeting IL-6 would help prevent or treat rheumatoid arthritis and other chronic inflammatory diseases [18]. iNOS is one of the key enzymes generating nitric oxide (NO), which regulates a number of pathophysiological conditions including infection, inflammation, and neoplastic diseases. Reagents that can inhibit iNOS would reduce the generation of NO and have potential anti-inflammatory effects [16]. Anther biomarker TNF-*α*, an adipokine, is involved in series of inflammation reactions during immune response. Its antagonists have also shown effects in the treatment of a range of inflammatory diseases, including rheumatoid arthritis, psoriasis, and ankylosing spondylitis [19].

To further explore the mechanism of antiproliferation and anti-inflammatory activities, extracts of* X. caffra* were evaluated in cells for suppression of NF-*κ*B (nuclear factor kappa-light-chain-enhancer of activated B cells), a protein complex that regulates the transcription of DNA and is involved in cellular responses to stress. The activation of NF-*κ*B is generally considered to upregulate a range of genes encoding growth factors, cytokines, apoptosis, and immunomodulatory molecules. As a shared key signal pathway of proliferation and inflammation in cells, the prolonged activation of NF-*κ*B has been reported to be involved in inflammatory conditions, autoimmune disease, and cancer [20–24]. The results demonstrate that the underlying therapeutic mechanism involves the suppression of NF-*κ*B, a shared pathway between cell death and inflammation.

## 2. Experimental

### 2.1. Materials

Standard compounds, gallic acid, rutin, catechin, quercetin, kaempferol, and ABTS (2,2′-azino-bis (3-ethylbenzthiazoline-6-sulfonic acid)) reagents were purchased from Sigma Aldrich (St. Louis, MO). HPLC-grade methanol (MeOH), acetonitrile (ACN), and formic acid were purchased from Fisher Scientific Co. (Fair Lawn, NJ). Folin-Ciocalteu's phenol reagent was purchased from MP Biomedicals (Solon, OH). The plant samples of* X. caffra* leaves were field-collected from Northeastern Namibia using a research permit from the Ministry of Environment and Tourism, Namibia. Voucher specimens were prepared and sent to the National herbarium at the National Botanical Research Institute (NBRI) where the collected plants were authenticated. The voucher specimens were also deposited in both the National Botanical Research Institute, Windhoek, Namibia, and the Chrysler Herbarium & Mycological Collection, Biological Sciences Building, Rutgers University, New Brunswick, NJ. The leaves were manually harvested using NBRI guidelines and were air-dried under ambient temperature for two weeks and then packaged for later experimental analysis. The dried leaf materials were air-shipped to Rutgers University where each was ground into a fine powder with a laboratory mill (Perten 3100) and stored at room temperature in the dark.

### 2.2. Analytical Equipment

Separation on HPLC was performed using column Prodigy ODS3 5 *μ*m, 150 × 3.2 mm, 5 micro (Phenomenex Inc., Torrance, CA). The separation, identification, and quantification were performed on Hewlett-Packard Agilent 1100 series HPLC-MS (Agilent Technologies, Waldbronn, Germany) equipped with a quaternary pump system, a degasser, an autosampler, a DAD detector, an MSD trap with an electrospray ion source (ESI), and software HP ChemStation, Data Analysis 4.2.

### 2.3. LC/MS Conditions for Qualification and Quantification of the Polyphenols

HPLC separation was performed with mobile phase of solvents A and B in gradient, where A was 0.1% formic acid (v/v) in water and B was 0.1% formic acid (v/v) in acetonitrile. The gradient starts from 5% B to 30% B in 50 min. The detection wavelength was set at 280 nm for gallic acid and catechin and 370 nm for quercetin and kaempferol derivatives. The flow rate is 1.0 mL/min. The electrospray was conducted in negative and positive modes in two separated runs. The optimized collision energy is of 80%, scanning from* m/z* 100 to 700. ESI was conducted at voltage of 3.5 kV for positive model and −3.5 kV for negative model. High-purity liquid nitrogen 99.999% was used as dry gas and nebulizer at a flow rate of 12 L/min, and the capillary temperature was 350°C. Nitrogen was used as nebulizer at 60 psi and helium as collision gas. The ESI interface and mass spectrometer parameters were optimized to obtain maximum sensitivity.

### 2.4. Sample Preparation

For quantitative and qualitative study, ~500 mg of the finely grinded plant material was extracted using 10 mL 70% methanol in water with 0.1% acetic acid and sonicated for 10 min. Then, the extract was conditioned at room temperature for overnight and filtered through a 0.45 *μ*m filter. 10 *μ*L of the extract was injected for both qualitative and quantitative analyses. The extraction procedure was adopted from our prior studies [25]. For total phenolic assay,* in vitro* antioxidant assay: around 20 mg of each plant sample was extracted using 10 mL 70% methanol/water (0.1% acetic acid) followed by sonication for 10 min and then conditioned at room temperature for overnight. The extract was filtered through a 0.45 *μ*m nylon filter. For cell-based bioactivity assays, the leaf extract above was dried on rotavapor and lyophilizer to powder and subsequently dissolved in DMSO to different concentrations.

Individual stock solution (~1.0 mg/mL) of the standard compound was prepared by dissolving ~10.0 mg of each compound in 10 mL methanol. The stock solution was diluted with methanol to obtain working solutions ranging from 3.91 to 500 *μ*g/mL. Calibration curve was built upon each group of the working solutions. In each standard curve, six or more concentration levels were used for calibration on HPLC. The concentration of quercetin (*y* = 0.052*x* − 0.8257, *R* ^2^ = 0.9999) and kaempferol (*y* = 0.0383*x* − 1.7818, *R* ^2^ = 0.9999) ranges from 3.91 to 500 *μ*g/mL. For gallic acid (*y* = 0.0373*x* − 12.437, *R* ^2^ = 0.9999) and catechin (*y* = 0.1516*x* − 3.5055, *R* ^2^ = 0.9999), the concentration ranges from 31.25 to 500 *μ*g/mL. All calibrations curves have good linearity.

### 2.5. Cell Culture and Treatment

The murine RAW 264.7 macrophage cell, a widely used model for inflammatory studies, was obtained from the American Type Culture Collection (ATCC). PC-3/N cell, a stable clone transfected with an NF-*κ*B luciferase construct, was established and obtained from Dr. Xi Zheng's laboratory as previously described [26]. The RAW cell and PC-3/N cell were routinely cultured in Dulbecco's Modified Eagle's Medium (DMEM, Invitrogen, Carlsbad, CA, USA) supplemented with 10% fetal bovine serum (FBS, Invitrogen, Carlsbad, CA, USA), 100 U/mL penicillin, and streptomycin in a humidified incubator with 5%  CO_2_  at 37°C.

### 2.6. Folin-Ciocalteu Assay

Total phenolic content was measured on UV-Vis spectrophotometer according to the Folin-Ciocalteu's method [27]. 40 *μ*L of the extract was mixed with 900 *μ*L Folin-Ciocalteu's reagent followed by incubation at room temperature for 5 min. After that 400 *μ*L of 15% sodium carbonate was added and the mixture stayed at ambient room temperature for 45 min. Then, the UV absorption at wavelength of 752 nm was measured against a blank solution. The standard curve was measured based on the prepared gallic acid standard solution (1.0, 0.5, 0.25, 0.125, and 0.0625 mg/mL) and the result was transformed as milligrams of gallic acid per gram of sample ± SD.

### 2.7. ABTS Scavenging Assay

The total antioxidant capacity of leaf extract was measured according to the decolorization of the ABTS radical cation as percentage of inhibition. ~38.4 mg of ABTS and 6.6 mg potassium persulfate (K_2_S_2_O_8_) were codissolved in 10 mL of water and reacted for 16 hours in dark environment to form stable radicals. The ABTS working solution was prepared by dissolving ABTS radicalized solution in ethanol to an absorbance of 0.700 ± 0.20 at *λ* = 734 nm. The wavelength selected is corresponding to the highest extinction coefficient (*ε* = 1.7 × 10^4^ mol^−1^L cm^−1^ in ethanol). 990 *μ*L of the diluted ABTS radical solution was mixed with 10 *μ*L of the sample extract followed by reaction for 20 minutes at room temperature. The decoloration of the solution indicates that ABTS radical cations were reduced by the antioxidants in the sample and was determined by the measurement of the decrease of absorbance at 734 nm against a blank solution of ethanol at 25°C. The percentage decrease of absorbance at 734 nm has a linear relationship with the amount of antioxidant compounds in the extract and can be calculated using the following equation:

(1) percentage  inhibition  (%)  =1−AbsorbancesampleAbsorbancecontrol×100.

Standard solution of Trolox was prepared in ethanol. The linear relationship was built up between decreased absorbance percentage and Trolox concentration (*r* ^2^ = 0.99). All samples are prepared in triplicate and the result was expressed as mmol of Trolox per gram of sample ± SD.

### 2.8. Antiproliferation Assay

The antiproliferative effect of* X. caffra* leaf extract was tested in RAW cells using the MTS assay. MTS assay, usually called “one step” MTT assay, is used for assessing cell viability. NAD(P)H-dependent cellular oxidoreductase enzymes from live cells can reduce MTS reagent and form a formazan product with maximum absorption at 490–500 nm. The RAW cells were seeded in a 96-well tissue culture plate at an initial density of 10,000 cells/mL. After 24 hr, the cells were treated with various concentrations of* X. caffra* leaf extract in DMSO for another 24 hr. The MTS assay was then performed using the CellTiter 96 aqueous nonradioactive cell proliferation assay kit [MTS: 3-(4,5-dimethylthiazol-2-yl)-5-(3-carboxymethoxyphenyl)-2-(4-sulfophenyl)-2H-tetrazolium, inner salt] (Promega, Madison, WI, USA), as previously described [24, 28]. The absorbance of the formazan product was measured at 490 nm using a *μ*Quant Biomolecular Spectrophotometer (Bio-Tek Instruments, Inc., Winooski, VT, USA). The cell viability (%) was expressed as follows: (optical density of sample)/(optical density of DMSO) × 100%.

### 2.9. Quantitative Real-Time Polymerase Chain Reaction (qPCR)

The RAW cells were seeded in 6-well tissue culture plates at the density of 10,000 cells/mL. After 24 hours, the cells were treated with LPS (1 *μ*g/mL, Sigma, St. Louis, MO, USA) alone or cotreated with the leaf extract (78.13–312.5 *μ*g/mL) dissolved in DMSO. Then, RAW cells were harvested 24 hours after each treatment. Total RNA was isolated and purified using RNeasy Mini Kit (Qiagen, Valencia, CA, USA) according to the manufacturer's manual. The cDNA was synthesized from 1 *μ*g of total RNA by using the TaqMan Reverse Transcription Reagents (Applied Biosystems, Foster City, CA, USA). The gene expression of IL-6, iNOS, and TNF-*α* was quantitated by qPCR performed on an Applied Biosystems 7900HT Fast Real-Time PCR System (Applied Biosystems, Foster City, CA, USA) with SYBR Green PCR Master Mix. The primer sequences were described previously [29].

### 2.10. Luciferase Assay of NF-*κ*B-Dependent Reporter Gene Activity

The PC-3/N cells were seeded in 12-well tissue culture plates at an initial density of 10,000 cells/mL. After 24 hours, the cells were treated with the leaf extract (78.13–312.5 *μ*g/mL) dissolved in DMSO. Then, RAW cells were harvested 24 hours after each treatment. The luciferase activity was measured using a luciferase reporter assay system (Promega, Madison, WI, USA) according to the manufacturer's manual. Briefly, the treated PC-3/N cells were washed twice with ice-cold phosphate-buffered saline (1X PBS, pH 7.4) and harvested in 1X reporter lysis buffer. After centrifugation at 12,000 rpm for 5 min at 4°C, a 10 *μ*L aliquot of the supernatants was mixed with 50 *μ*L of luciferase assay substrate and the mixture was assayed for luciferase activity using a SIRIUS luminometer (Berthold Detection System GmbH, Pforzheim, Germany). After normalized with protein concentration detected by BCA protein assay (Pierce, Rockford, IL, USA), NF-*κ*B luciferase activity was expressed as percent of the control.

## 3. Results and Discussion

### 3.1. Identification of Phytochemical Constituents

**Figure 1:**
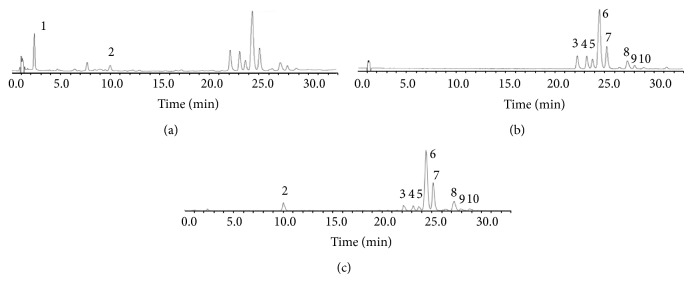
Chromatograms of* Ximenia caffra* leaf extract. (a) UV chromatogram at 280 nm; (b) UV chromatogram at 370 nm; (c) processed MS chromatogram.

Identification of each compound was mainly based on the mass spectrometric data, UV-Vis spectrum, and comparison with authentic standards. Each compound was analyzed on mass spectrometry under both negative and positive modes. The UV and MS chromatograms are illustrated in Figure 1. The identity, retention time, molecular ions, fragment ions, and maximum UV absorption wavelength for each identity are summarized in Table 1.

**Figure 2:**
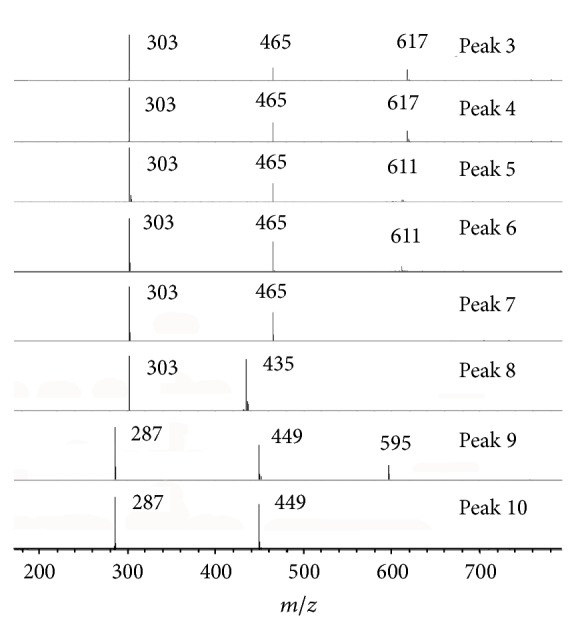
The mass spectra of flavonol glycosides (peak 3 to peak 10) under positive ion mode.

**Figure 3:**
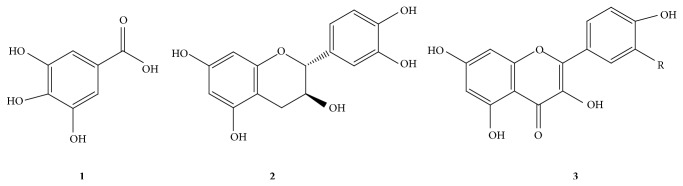
Representative structures of identified polyphenols. (**1**) Gallic acid; (**2**) Catechin. (**3**) The aglycone of compound** 3**–**10**: quercetin (R=OH) and kaempferol (R=H). Sugar moieties are usually attached at position 3.

The major compounds (**3**–**10**) detected in* X. caffra* leaf are flavonol glycosides. Compounds** 3**–**10** were identified as glycosylated derivatives of quercetin and kaempferol based on their characteristic aglycone fragment ions (*m/z* = 303,* m/z* = 287 under positive ionization). The main glycosides attached are galactoside/glucoside, xyloside, and rutinoside. The UV-Vis absorption of these identified compounds has a maximum absorption at ~356 nm, which is consistent with previous reports [30]. The structure of each derivative was identified based on the molecular and fragment ions. For example, MS spectrum of compound** 6** (*t*_*R*_ 24.3 min) indicates that it has molecular ions at* m/z* 611 ([M + H]^+^) and* m/z* 609 ([M − H]^−^) and it is fragmented to* m/z* 465 [M − rhamnosyl + H]^+^ and* m/z* 303 ([M − rhamnosyl − glucosyl + H]^+^), which corresponds to quercetin-rutinoside, and the identity was further confirmed with commercially available authentic standard. The same identification was applied to the derivatives of galloylglucoside/galloylgalactoside. For example, compound** 3**/**4** (*t*_*R*_ 22.1 min or *t*_*R*_ 23.1 min) has a molecular ion at* m/z* 617 ([M + H]^+^) and it is further fragmented to* m/z* 465 ([M − galloyl + H]^+^) and* m/z* 303 ([M − galloylglucoside + H]^+^ or [M − galloylgalactoside + H]^+^). The MS spectra of the flavonol glycosides under positive ion mode are illustrated in Figure 2 and the structures of representative compounds are shown in Figure 3.

Some components give the same MS, UV-Vis spectrum, such as compound** 3**/**4** and** 5**/**6**. Compounds** 3** and** 4** may have different glycosides (galactoside or glucoside) attached, or same glycosides but at different positions. Compound** 6** can be differentiated from** 5** based on a comparison with commercial available standard and can be determined as quercetin-3-O-rutinoside.

Other polyphenol compounds including gallic acid and catechin were also determined. Their detection under negative mode yields better sensitivity. Gallic acid has a molecular ion at* m/z* 169 ([M − H]^−^) and a fragment ion at* m/z* 125 ([M − CO_2_ − H]^−^). Catechin has a molecular ion at* m/z* 289 ([M − H]^−^). Both have characteristic maximum absorption at ~280 nm. To further confirm the identities of gallic acid and catechin, each sample was coinjected with respective authentic standard.

### 3.2. Quantification of Each Phytochemical

The leaves of* X. caffra* were found to contain copious amounts of different flavonol glycosides and other polyphenol compounds. The contents of flavonol glycoside products were quantified at UV wavelength of 370 nm. To approximate the content of compounds without commercially available standards, the response factor was used and adjusted by the ratio of the molecular weight to that of the aglycone [31]. Gallic acid and catechin were quantified at 280 nm. The content of each individual polyphenol is included in Table 1. The total amount of polyphenols measured by HPLC is 19.46 mg/g, 81% of which is contributed by quercetin and its derivatives (15.64 mg/g), while kaempferol derivatives represent only 5% of the total amount. The most abundant single compound is rutin (9.08 mg/g), accounting for 47% of the total composition. Gallic acid, a phenolic acid, is 0.95 mg/g and represents 5% of the total amount. The only flavanol compound catechin (1.77 mg/g) contributes 9% of the total composition.

### 3.3. Total Phenolics and* In Vitro* Antioxidant Capacities

The content of phenolics in the sample is expressed as mg of gallic acid equivalents (GAE) per gram of ground powder. The total content of phenolics determined by Folin-Ciocalteu assay in the leaf is 261.87 ± 7.11 mg GAE/g. The antioxidant capacity of the samples is indicated using mmol of Trolox equivalents per gram of ground plant material. The total antioxidant capacity of the leaf extract determined by ABTS radical cation is 1.46 ± 0.01 mmol Trolox/g. It has been widely accepted that polyphenol compounds can reduce radicals* in vitro *and* in vivo*, so it is highly possible that this activity is due to its content of flavonoids described above.

### 3.4. Antiproliferation Analysis

**Figure 4:**
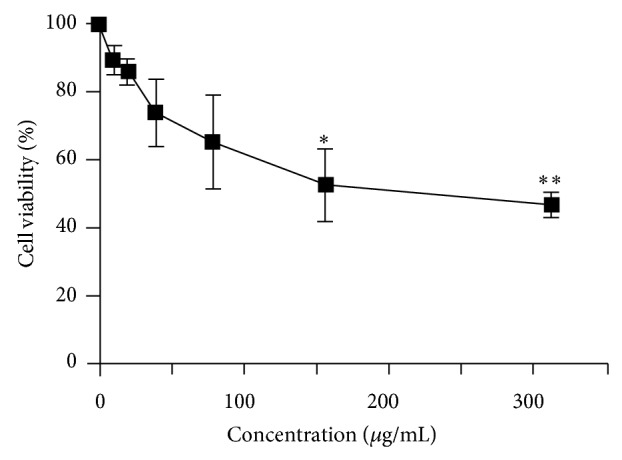
Cell viability versus the concentrations of leaf extract measured by MTS assay.

The MTS assay was applied to investigate the effect of* X. caffra* leaf extract on the growth of cultured RAW 264.7 cells. As illustrated in Figure 4, after 24 hours of treatment with leaf extract, the cell viability of RAW cells decreased the dose dependently. When the concentration of raw leaf extract is 312.5 *μ*g/mL, the viability of the RAW cell is 46.83 ± 3.46%. The IC_50_ value of the raw leaf extract was determined to be 239.0 ± 44.5 *μ*g/mL. This data implies that the leaf extract inhibits the growth of RAW cell, potentially possessing a cytotoxic effect.

### 3.5. Anti-Inflammatory Analysis

**Figure 5:**
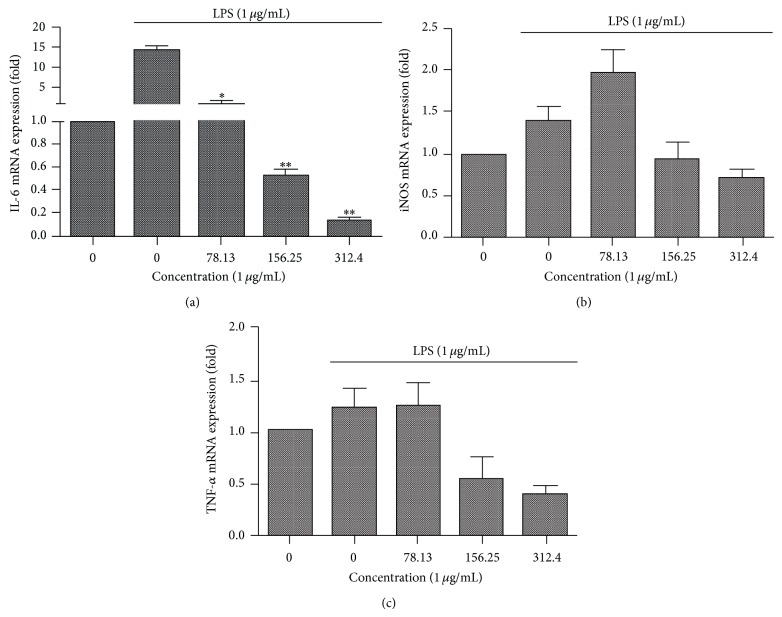
Anti-inflammatory activity of* Ximenia caffra* leaf extract measured by RT-qPCR in RAW 264.7 macrophage cells (a) IL-6 mRNA expressions after treatment; (b) iNOS mRNA expression after treatment; (c) TNF-*α* mRNA expression after treatment.

To evaluate the effect of* X. caffra* leaf extract on the LPS-stimulated expression of inflammatory enzymes and proinflammatory cytokines, the mRNA expressions of inflammatory markers IL-6, iNOS, and TNF-*α* were measured by qPCR. Treatment of cells with leaf extract at a variety of concentrations demonstrated a dose-dependent response on the expression of IL-6, iNOS, and TNF-*α*, as shown in Figure 5. The strongest response was seen for IL-6, (Figure 5(a)) wherein treatment at 312.5 *μ*g/mL induced a nearly 10-fold decrease in expression versus background expression and nearly 100-fold decrease in expression versus LPS induced cells which were not treated by* X. caffra* leaf extract. This demonstrates clearly the impact of* X. caffra* leaf extract on the expression of IL-6, an indication of potentially significant anti-inflammatory activity. The effect of* X. caffra* extract on TNF-*α* and iNOS was measurable but less significant than the results for IL-6. The expression of iNOS in cells treated with 312.5 *μ*g/mL leaf extract was comparable to background expression, a twofold decrease in expression compared to LPS induced cells (Figure 5(b)). A twofold decrease in the expression of TNF-*α* was measured in cells treated with 312.5 *μ*g/mL when compared to background expression, which is a threefold decrease in the expression compared to LPS induced cells that were not treated with* X. caffra* extract (Figure 5(c)). These results corroborate well with IL-6 results, further supporting the anti-inflammatory activity of* X. caffra* leaf extracts in a cell system.

### 3.6. NF-*κ*B Transcription Activity

**Figure 6:**
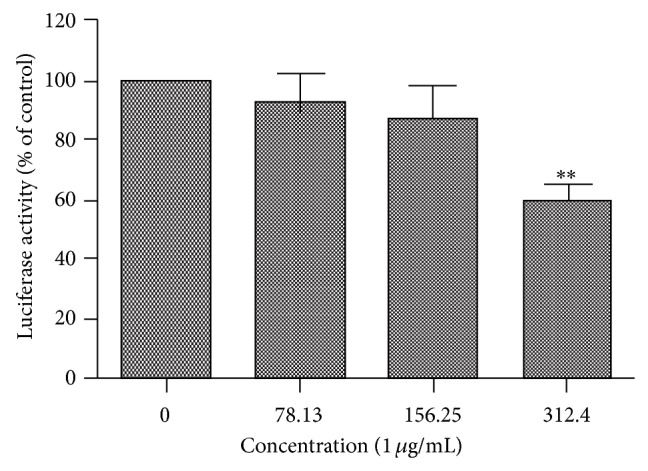
NF-*κ*B transcription activity versus the concentrations of* Ximenia caffra* leaf extract after treatment.

As described above, the leaf extract of* X. caffra* has shown both antiproliferation and anti-inflammatory activities in RAW cells. NF-*κ*B protein complex is involved in a number of cellular responses to stimuli such as free radical, stress, and cytokine [32, 33]. To investigate the effects of* X. caffra* leaf extract on the NF-*κ*B transcription activity, the luciferase activity of PC-3/N cells, with a stably transfected NF-*κ*B luciferase constructs, was measured. In this assay, protein NF-*κ*B was coexpressed with protein luciferase and directly quantified by luminometer. The data demonstrated that* X. caffra* leaf extract significantly attenuated the NF-*κ*B transcription activity in a dose-dependent manner after 24 h treatment (Figure 6). At the concentration of 312.5 *μ*g/mL, the expression of NF-*κ*B was decreased to approximately 60% of the vehicle control. The data obtained is consistent with the results from MTS and qPCR assays, indicating that the inhibition of NF-*κ*B activation might be one of the mechanisms underlying the antiproliferation and anti-inflammatory effects of the leaf extract of* X. caffra*.

## 4. Conclusion

This is the first study examining the leaves, rather than the fruit or seeds of* X. caffra*, which can be sustainably harvested but underutilized by local indigenous peoples. The chemical profile of* X. caffra* leaf was comprehensively analyzed and led to the identification of 10 polyphenol compounds, including phenolic acid and flavonoids. The individual polyphenols were successfully quantitated using UV detection. Further bioactivity investigations showed that the extracts of* X. caffra* leaf exhibit antioxidant, antiproliferation, and anti-inflammatory activities. The underlying molecular mechanism may partially be contributed by the inhibition of NF-*κ*B activation, a shared signal pathway between proliferation and inflammation. Further investigations are needed to explore whether these polyphenol compounds could work synergistically to achieve much improved activities than each single component. The chemical profile and bioactivities determined support its traditional use and may help for its further pharmacological studies and nutraceutical applications. Considering that some botanical supplements on market have similar polyphenol profile, the compounds found in* X. caffra* leaf would also be of interest as a new source of these natural products. The development of a sustainable production and collection system for* X. caffra* leaves could compliment interest in this plant's fruit and seeds.

## Figures and Tables

**Table 1:** Phytochemicals identified from the leaf extract of *Ximenia caffra*.

Peak	Retention time (min)	UV-Vis *λ*_max⁡_ (nm)	Molecular and fragment ions		Compound name	Content (mg/g)
			Negative ions	Positive ions		
1	2.4	278	125, 169	—	Gallic acid^*^	0.96
2	10.0	280	289	291	Catechin^*^	1.77
3	22.1	356	615	303, 465, 617	Quercetin-G-Gall	1.70
4	23.1	358	615	303, 465, 617	Quercetin-G-Gall	1.59
5	23.6	357	609	303, 465, 611	Quercetin-G-Rha	1.12
6	24.3	357	463, 609	303, 465, 611	Quercetin-Glc-Rha (Rutin)^*^	9.08
7	25.1	356	463	303, 465	Quercetin-G	2.03
8	27.2	356	433	303, 435	Quercetin-Xyl	0.11
9	27.9	356	593	287, 449, 595	Kaempferol-G-Rha	0.82
10	28.9	356	447	287, 449	Kaempferol-G	0.26

G: glucosyl/galactosyl; Glc: glucosyl; Gla: galactosyl; Rha: rhamnosyl; Gall: galloyl; Xyl: xylosyl; ∗ indicates that compounds were unambiguously confirmed through the comparison with authentic standards.
